# Thrombus Formation on Amplatzer Septal Occluder Device: Pinning Down the Cause

**DOI:** 10.1155/2014/457850

**Published:** 2014-09-02

**Authors:** Kevin Belgrave, Shaun Cardozo

**Affiliations:** Division of Cardiology, Department of Internal Medicine, Detroit Medical Center, Wayne State University, Detroit, MI 48201, USA

## Abstract

The use of interatrial septal occluder devices is an efficacious and less invasive alternative to open heart surgery for the repair of atrial septal defects. These devices present significant risks including thrombus formation on the device and subsequent thromboembolic events. We present a case of a woman who presented with stroke-like symptoms five years after PFO closure. The patient was subsequently found to have a thrombus on the occluder device. Our case highlights the risk of such thrombolic phenomenon and the risk associated with the device structure as a nidus for such a complication.

## 1. Introduction

The use of interatrial septal occluder devices has become an efficacious and less invasive alternative to open heart surgery for the repair of both atrial septal defects and patent foramen ovales (PFOs). Of the known complications, thrombus formation on the device and subsequent thromboembolic events are one of the most paramount concerns facing clinicians and interventionalists. Much data has been gathered on the predisposing factors that may contribute to such phenomenon underlying, namely, coagulopathies [1], age, female gender, relative safety of device (CardioSEAL (Nitinol Medical Technologies, Inc., Boston MA) versus Amplatzer (AGA Medical Corporation, Golden Valley, Minnesota), with Amplatzer having fewer thromboembolic events) [2], use of anticoagulant therapy, arrhythmias, atherosclerosis of the aorta, and duration of time since implantation (with most studies reporting events within the first year after implantation) [3]. We present a case of a woman who presented with stroke-like symptoms five years after PFO closure with an Amplatzer septal occluder device. The patient had no history of hypercoagulable states or other risk factors and was not currently on anticoagulation other than aspirin.

## 2. Case Report

Our patient is a 46-year-old left-handed Caucasian female with a history of migraines and a prior TIA with no residual deficits. The TIA occurred in 2007 and no embolic source was identified at the time. However, since a PFO on transthoracic echocardiogram (TTE) was discovered during the TIA event, this was subsequently closed in 2007 due to suspected paradoxical embolus. After PFO closure, the patient had the appropriate routine follow-up that included serial TTEs at the indicated intervals within the first year. She then presented five years later in 2012 to another facility with the acute onset of right sided weakness and severe aphasia. She received tPA and was transferred to our hospital where she underwent angiography that showed severe occlusion of the left M1 and inferior and superior M2 segments of the middle cerebral artery. She underwent thrombectomy of her right M1 with follow-up angiography showing TIMI 3 flow restoration in the left M1 and inferior M2 with only TIMI 1 flow restoration in the superior portion of M2, which remained partially occluded. A repeat CT head showed hemorrhagic transformation affecting the left lentiform nucleus and left caudate head. Aspirin was discontinued at that time. It is unclear from the medical records why the patient was on aspirin originally although it was suspected that it was instituted after PFO closure.

**Figure 1:**
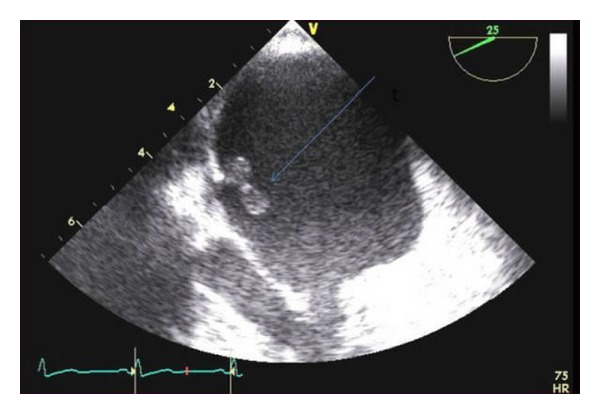
TEE view of thrombus in the left atrium attached to the pin of the Amplatzer septal occluder device.

Once the patient was stable and transferred from the Neuro-ICU to the neurology floor service, a transesophageal echocardiogram (TEE) was performed and demonstrated a normal ejection fraction and visualized a well seated, stable, and Amplatzer septal occluder device. There was also visualization of a mobile thrombus attached to an occluder pin on the left atrial disc side of the device. The thrombus measured 0.5 cm by 1.2 cm at its greatest dimension (Figure 1). There was no residual flow across the occluder device. The recommendation from cardiology, after discovering this thrombus, was for lifelong anticoagulation with Coumadin.

While in the ICU, patient began regaining capacity to verbalize, although expressive aphasia remained severe. Her strength improved to near baseline on the affected side following thrombectomy. Young stroke work-up labs available at the time of discharge (i.e., ANA, ENA, lupus anticoagulant, and proteins C and S) were all normal or negative. No further workup was done and the patient was discharged home in stable health with appropriate outpatient followup.

## 3. Discussion

Although it is a well-known and dreaded complication, thromboembolism remains an exceedingly rare phenomenon postseptal occluder device implantation. However, when seen, as with our patient, the effects can be life-altering or even fatal. Studies have shown that most thromboembolic events and associated neurological sequelae occur within the first year after implantation. The recommendation from the device manufacturers is to perform an echocardiogram one week, six months, and one year after implantation. The surveillance echocardiograms done at the recommended intervals within one year did not demonstrate any thrombus formation on our patient. However, our patient developed this thromboembolic stroke with residual neurologic deficits 5 years later. There is some data to suggest that these events are related, at least in part, to residual flow across the defect [4]. Despite nearly equal incidence in residual shunt in patients, the Amplatzer had far fewer thromboembolic events than its counterpart the CardioSEAL septal occluder device. In fact, some studies demonstrate the occurrence with the Amplatzer to be quite rare. Our patient was outfitted with the Amplatzer device and had no residual flow. Of the major risk factors, there only remains a possible underlying coagulopathy.

The patient has no history of a coagulopathy and was screened for thrombogenic processes prior to discharge with all studies being within normal ranges or negative. This patient's unfortunate complication to the procedure proved to be quite rare in that, of the devices available in the United States, she was implanted with the one associated with least thrombotic risk. One study compared the 3 main occluder devices and found higher rates of thrombus formation with the CardioSEAL-STARflex than the Helex or Amplatzer [5]. In that study, a total of 12 patients developed device thrombus formation (11 with the CardioSEAL-STARflex and 1 with the Helex). All those events occurred within the first year of device implantation. Therefore, the complication of thrombus formation on the Amplatzer device is rare and its occurrence years after implantation is unusual. Of the other associated risks, she presented with only age and gender. It remains to be seen whether there is any role here for surveillance imaging or other modalities to periodically reevaluate the device and risk for thromboembolism past the normal one year follow-up.

Also, although the device itself is more thrombogenic, the future design of closure devices may need altering to correct other niduses for thrombus formation like the central pin.
